# Developing Seedless Growth of ZnO Micro/Nanowire Arrays towards ZnO/FeS_2_/CuI P-I-N Photodiode Application

**DOI:** 10.1038/srep11377

**Published:** 2015-06-16

**Authors:** Zhi Yang, Minqiang Wang, Sudhanshu Shukla, Yue Zhu, Jianping Deng, Hu Ge, Xingzhi Wang, Qihua Xiong

**Affiliations:** 1 Electronic Materials Research Laboratory (EMRL), Key Laboratory of Education Ministry; International Center for Dielectric Research, Xi’an Jiaotong University, Xi’an 710049, China; 2 Division of Physics and Applied Physics, School of Physical and Mathematical Sciences, Nanyang Technological University, Singapore 637371, Singapore; 3 Energy Research Institute, Interdisciplinary Graduate School, School of Materials Science and Engineering, Nanyang Technological University, Singapore 639798, Singapore; 4 NOVITAS, Nanoelectronics Center of Excellence, School of Electrical and Electronic Engineering, Nanyang Technological University, Singapore 639798, Singapore

## Abstract

A seedless hydrothermal method is developed to grow high density and vertically aligned ZnO micro/nanowire arrays with low defect density on metal films under the saturated nutrition solution. In particular, the mechanism of seedless method is discussed here. A buffer layer can be confirmed by transmission electron microscopy (TEM), which may release the elastic strain between ZnO and substrate to achieve this highly mismatched heteroepitaxial structures. Based on ZnO micro/nanowire arrays with excellent wettability surface, we prepared ZnO-FeS_2_-CuI p-i-n photodiode by all-solution processed method with the high rectifying ratio of 197 at ±1 V. Under AM 1.5 condition, the J_sc_ of 0.5 mA/cm^2^, on-off current ratio of 371 and fast photoresponse at zero bias voltage were obtained. This good performance comes from excellent collection ability of photogenerated electrons and holes due to the increased depletion layer width for p-i-n structure. Finally, the high responsivity around 900 nm shows the potential as near infrared photodetectors applications.

As a promising n-type semiconductor with wide direct bandgap of 3.37 eV, high electron mobility, and good thermal and air stability^1^, zinc oxide (ZnO) has been widely used as electron transport / hole blocking layer in optoelectronic devices such as photovoltaics and photodetectors^2^,^3^,^4^,^5^. ZnO nanowires (NWs) can be grown on a variety of substrates such as ZnO bulk single crystals, silicon, ITO glass, GaAs, sapphire, metal thin films, graphene and flexible substrates by chemical bath deposition (CBD)^6^,^7^,^8^, which is a low-temperature and low-cost technique compared with chemical vapor deposition (CVD) and other physical deposition methods. However, this method usually requires preparing ZnO seeds layer (so-called buffer layer) first to relax the strain between NWs and substrates in highly mismatched heteroepitaxial structures. This buffer layer can act as the container of strain from the lattice mismatch^9^. Then the growth of the NWs array on the buffer layer can be regarded as homoepitaxial growth. On the contrary, seedless and catalyst-free growth of ZnO NWs arrays can be more attractive due to simple operation and new mechanism. Van der Waals epitaxy (vdWE) has been developed to obtain many defect-free nanostructures such as single-crystalline films^10^, nanosheets^11^,^12^ and nanowires^13^,^14^ growing on substrates even with a large lattice mismatch. Recently, our group reported that ZnO wire arrays could be grown in a solution phase synthesis on mica substrates at low temperature based on vdWE mechanism, and the very low deep level emission indicated their low defect density^15^. However, this method requires vdWE substrate such as mica, which is usually nonconductive preventing the direct applications in optoelectronic devices without transferring process. Here in this report, we further develop a seedless synthesis of ZnO micro/nanowire with controllable density on metal thin films directly using a hydrothermal approach. The metal thin films can not only act as the nucleation sites to assist the growth of ZnO micro/nanowire, but also serve as the bottom electrode in device fabrications. Compared with previous similar seedless methods^16^,^17^,^18^, our results show that the vertically aligned ZnO micro/nanowire with high density in a large coverage of 1 × 1 cm^2^ can be obtained from saturated nutrition solution. Many parameters including concentration of nutrition solution, growth time, and substrate-dependent are studied in detail. In particular, the mechanism of seedless method is discussed based on TEM image of interface between Au and ZnO, and evolution of ZnO micro/nanowire arrays. Besides, compared with previous report^19^ on seedless growth of ZnO via galvanic-cell approach, making use of the contact potential between different materials as the driving force for ZnO growth, our seedless method is easier without any sacrificing layer. Moreover, compared with traditional polycrystalline ZnO films as an electron transporting layer, this vertically aligned ZnO micro/nanowire arrays with high density have orientation, resulting in better electron transporting ability along the direction of micro/nanowire. At the same time, the porous top of 1D ZnO micro/nanowire arrays can be used to build 3D heterojunction configuration, employing their large surface area to improve heterojunction interfacial area. The compact bottom of ZnO micro/nanowire arrays can hinder infiltrating p-type materials to contact with the bottom electrode. So the ZnO micro/nanowire arrays by this seedless method have dual functions.

Pyrite iron disulfide (FeS_2_) has attracted people’s attention recently due to its large optical absorption coefficient (>10^5^ cm^−1^), narrow band gap of 0.95 eV, and the advantages of abundance and non-toxicity^20^,^21^. These unique properties make it as a promising candidate for solar cells, photodetectors and counter electrode materials^22^,^23^,^24^,^25^. Even though, the rectification ratio of photodiode utilizing FeS_2_ as a light absorber is limited due to large leakage current^3^,^26^, and low power conversion efficiency of solar cell is from low open-circuit voltage due to sulfur deficiencies and surface states^20^. Some measures were adopted to overcome these challenges. For example, Wang *et al.* used a high work function MoO_3_ thin film as electro-blocking layer to avoid leakage current effectively, resulting in large on-off current ratio^3^. Gong *et al.* created FeS_2_/CdS photodiode with high rectification ratio and demonstrated photodiode utilizing FeS_2_ quantum dots (QDs) had lower leakage current compared with nanocubes with [111] terminated^24^. Copper iodide (CuI) as an inorganic p-type semiconductor has wide bandgap, high p-type conductivity, good solution-processable and low-cost, making it suitable to replace spiro-OMeTAD in perovskite and polymer solar cells^27^,^28^. So CuI was chosen here as an effective hole transport layer on the basis of suitable band position, and high hole conductivity.

The performance of FeS_2_ heterostructure devices can be improved from two aspects: one is the purity, surface, physical and chemical properties of material itself; the other is structure, band alignment and compatibility of each layer. At present, we are working on passivating surface state of FeS_2_ by halide treatment. Here, combining the advantage of vertically aligned ZnO micro/nanowire arrays by seedless method, we focus on the latter by building p-i-n heterostructure. For p-i-n heterostructure, the depletion-layer width is essentially fixed by the thickness of intrinsic layer, which does not vary significantly with bias voltages. The increasing depletion width can not only create greater light absorption but also reduce the junction capacitance for faster photoresponse, while not decrease the charge collection efficiency^29^. This is a unique advantage for p-i-n heterostructures compared with p-n heterostructures. There are many previous reports to confirm the effectiveness of p-i-n architecture, such as ZnO-PbSe-CuI p-i-n solar cell^30^, Si QDs p-i-n solar cell^31^, Si nanowires-based p-i-n photodetector^32^, GaAs nanowire array solar cell with axial p-i-n junction^33^ and P3HT-blend-PCBM p-i-n thin film photoconductors^34^. Here in this paper, we use n-type ZnO micro/nanowire arrays as electron transport layer, FeS_2_ nanocrystals (NCs) film as intrinsic light absorber layer and p-type CuI film as hole transport layer to form p-i-n heterostructure, and improving responsivity of FeS_2_ photodiode are confirmed.

## Results and Discussion

### Seedless growth of ZnO

**Figure 1:**
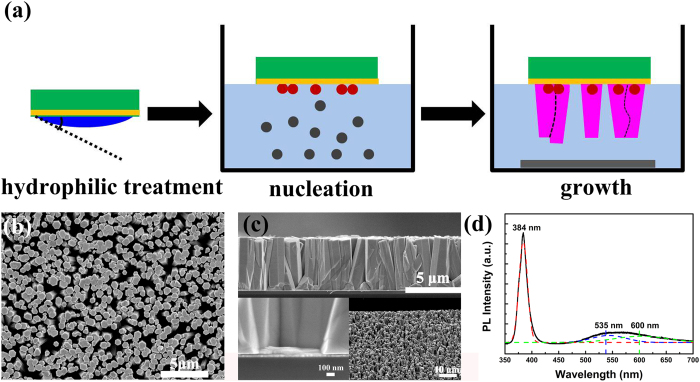
(**a**) Schematic diagram depicted seedless hydrothermal growth process of ZnO micro/nanowire arrays grown on Au film substrate. SEM images of ZnO micro/nanowire arrays on Au film with nutrition solution concentration of 80 mM: (**b**) plane view, (**c**) cross section, the left inset is magnified view of interface between ZnO and Au film, and the right inset is 60° view of ZnO arrays. (**d**) PL spectrum of ZnO micro/nanowire arrays at room temperature, and the dot lines are Gaussian fitting curves.

The seedless hydrothermal growth process with Au film substrate is depicted in Fig. 1a. Firstly, the good hydrophilic surface of Au film could be obtained. With the increasing temperature, the hydrolysis of Zn(NO_3_)_2_ provided Zn^2+^ and hexamethylenetetramine (HMTA) could slowly hydrolyze in the water solution to produce OH^-^ gradually^35^. Then the Au film surface could be served as nucleation sites when substrate was floated on the nutrition solution. The number of nuclei was proportional to the concentration of nutrition solution. After the nucleation, the newly arrived ions could only be used to the growth of ZnO micro/nanowire arrays because the critical size for a nucleus to grow into crystal was required and they had a bigger chance to reach the existing NWs than form the new nuclei on substrate^36^. So the concentration of nutrition solution determined the final ZnO micro/nanowire arrays density, which could be seen from Figure S1. In addition, when the number of nuclei was low, both the lateral growth and vertical growth could happen. With the increasing amount of nucleation, the lateral growth was suppressed. This physical confinement could be helpful to achieve vertical growth. From the scanning electron microscopy (SEM) of Fig. 1b and c, when the saturated nutrition solution is used, the vertically aligned hexagonal ZnO micro/nanowire arrays with high density can be obtained. The average diameter is about 600 nm and the length is 5 μm. Besides, a lot of pores seen from top view of ZnO micro/nanowire arrays provide the enough coating space for the next to infiltrate FeS_2_ NCs.

**Figure 2:**
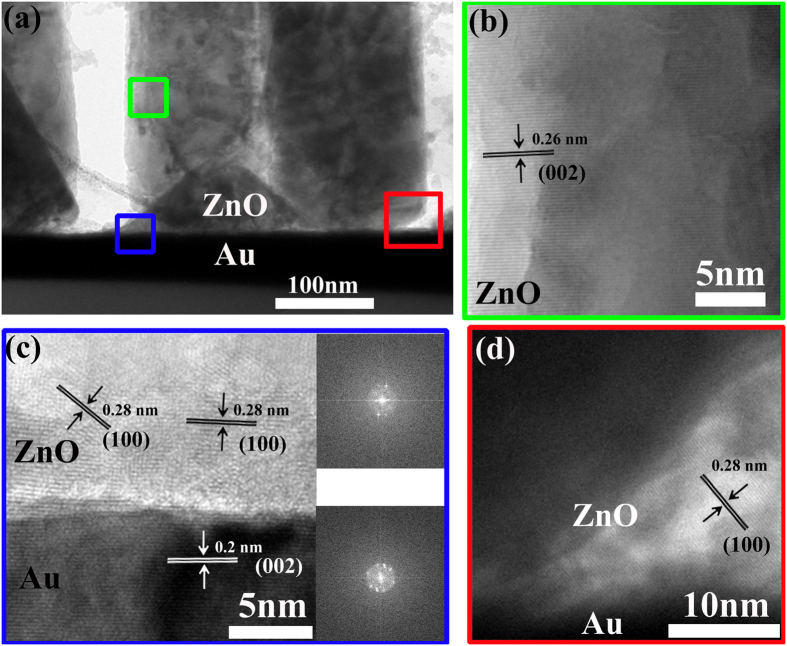
(**a**) Cross-sectional TEM image of ZnO micro/nanowire arrays on Au film. HRTEM images of the region as indicated by the corresponding colored squares, the insets in (**c**) are selected area electron diffraction image of corresponding region. The spacing between adjacent lattice planes are 0.26 nm and 0.28 nm, in agreement with the distance between two (002) and (100) crystal planes of wurtzite ZnO, respectively.

Next, we will discuss how the ZnO micro/nanowire arrays can grow on metal films by this heteroepitaxial seedless method. From magnified view of interface between ZnO micro/nanowire arrays and Au film in Fig. 1c and single ZnO micro/nanowire in Figure S2b, we can see a thin film between ZnO and Au film, which may come from ZnO nucleation layer. We infer that this thin film acts as buffer layer to release the elastic strain resulted from the lattice mismatch between ZnO and Au film. However, this buffer layer is often polycrystal and rich in crystalline defects, which allows releasing the strain of the system. Thus, the top segment of the NWs above the buffer layer can continue to grow without strain and defects^9^. The photoluminescence (PL) spectrum was collected to analyze the defect states of ZnO micro/nanowire arrays. From Fig. 1d, a strong and narrow UV emission peak at 384 nm can be assigned to the near band-edge emission, and the weak and broad emission in the visible region is ascribed to the defect emission. Based on the fitting results, the peaks at 535 nm and 600 nm are associated to oxygen vacancies and interstitial oxygen or hydroxide groups, respectively^37^. Compared with strong green emission using traditional methods with seeds^38^,^39^, the large ratio between UV and green emission indicates very low defect density of ZnO micro/nanowire arrays grown by seedless method without any atmospheric annealing treatments, which is in accordance with other report^40^. Furthermore, the TEM was performed to see the interface between Au and ZnO clearly, which can help us understand the initial growth process by this seedless method better. From Fig. 2a, we can see vertically grown ZnO nanowires on Au film. Based on high-resolution TEM (HRTEM) images in Fig. 2b, the upper single-crystal ZnO nanowire grows along the (002) direction. However, from Fig. 2c,d, we find that the ZnO at two interfaces are polycrystal and have (100) direction, indicating the different crystallinity between upper and bottom ZnO. Thus, we infer that the ZnO at the interface is so-called buffer layer formed at the nucleation stage. It is impossible to achieve epitaxal grown ZnO on Au directly due to the large lattice mismatch. So we believe that the ZnO at the interface in this case can play the role in releasing the elastic strain at the interface. Then upper ZnO can grow on this buffer layer based on homoepitaxial growth. In order to understand the growth process by this seedless method, the evolution of ZnO micro/nanowire arrays was investigated in Figure S3. The results indicate that the diameter and length of ZnO micro/nanowire arrays increase gradually with the time, and nucleation sites are critical at the beginning. Besides, the Au film could be replaced with Ag film, graphene, FTO and ITO glass in Figure S4. However, the diameter and aligned property of ZnO micro/nanowire arrays are different, which is explained based on surface wettablity and nucleation energy of different substrates in Supporting Information. In all, this seedless method can grow high density and vertically aligned ZnO micro/nanowire arrays with low defect density on metal films more easily.

### P-I-N heterojunction

**Figure 3:**
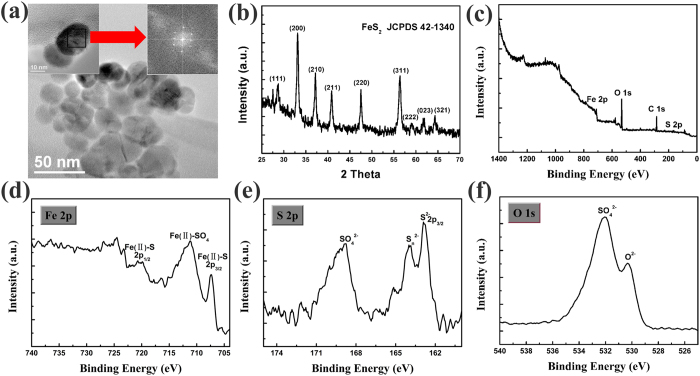
(**a**) TEM images of FeS_2_ NCs, the average size is 20 nm. The insets show HRTEM images of FeS_2_ NCs, and clear selected area electron diffraction image indicates single crystal of FeS_2_ NCs. (**b**) XRD of FeS_2_ NCs film. (**c**-**f**) XPS spectrum of FeS_2_ NCs film.

For an ideal p-i-n heterojunction, both of homogeneous layer should be highly doped to establish a strong electric field inside the intrinsic absorber layer^30^. ZnO NWs arrays usually have high electron concentration of 10^19^ cm^−3^ 41 and CuI film has a hole concentration of 7 × 10^17^ cm^−3^ 30. In our experiment, FeS_2_ NCs have average size of 20 nm and single-phased pyrite structure from X-ray diffraction (XRD) data shown in Fig. 3a,b. Besides, X-ray photoelectron spectroscopy(XPS) measurement was conducted to determine the elemental composition of the surface of FeS_2_ NCs. The general scan spectrum in Fig. 3c shows the presence of Fe 2p, O 1 s, C 1 s, and S 2p level. The Fe 2p spectrum in Fig. 3d has pyrite peaks of 2p_3/2_ at 707.3 eV and 2p_1/2_ at 720.2 eV. Besides, a strong peak at 711 eV is assigned to Fe (II)-SO_4_. The S 2p spectrum in Fig. 3e shows three sulfur species: pyrite S_2_^2-^ of 2p_3/2_ at 162.8 eV; S_n_^2-^ at 163.8 eV; SO_4_^2-^ at 168.7 eV. The O 1 s spectrum in Fig. 3f also shows the SO_4_^2-^ at 532 eV^42^,^43^. Therefore, the pyrite FeS_2_ is the dominant species, but the presence of SO_4_^2-^ indicates the outside FeS_2_ NCs convert to FeSO_4_ partially after air exposure. So the FeS_2_ NCs film used here is not strictly intrinsic layer. It has been observed that FeS_2_ thin films are usually p-type due to sulfur deficiencies and light oxygen doping which is reflected in the XPS analysis. The hole concentration of FeS_2_ NCs film is 10^18^ cm^−3^ 44,45, which is almost twice the concentration of CuI. However, the mobility of FeS_2_ NCs film is far lower than CuI film and ZnO micro/nanowire arrays. So the FeS_2_ NCs film layer can still be considered as intrinsic layer. Combined with band alignment discussed below, holes can diffuse from CuI film to FeS_2_ NCs film, while electrons can diffuse from ZnO micro/nanowire arrays to FeS_2_ NCs film in heterojunction. So the depletion region still can be created at the both interface sides of FeS_2_ NCs film.

**Figure 4:**
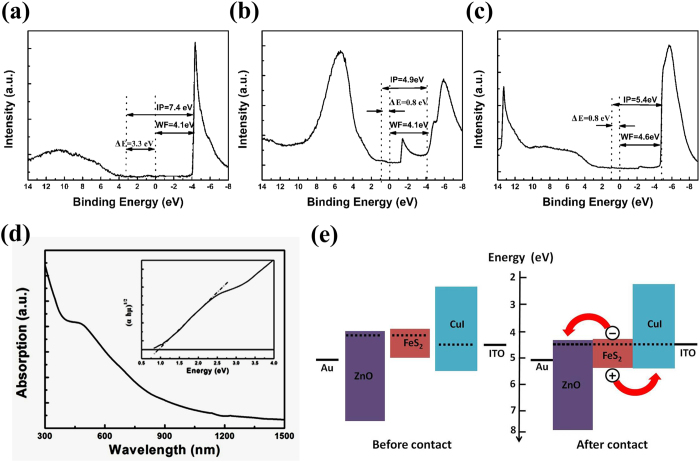
Valance photoemission and secondary electron spectrum of (**a**) ZnO micro/nanowire arrays, (**b**) FeS_2_ NCs film, (**c**) CuI film. The ionization potential (IP), work function (W_F_) and the difference ΔE between VBM and E_f_ are calculated. (**d**) Optical absorbance spectrum of FeS_2_ NCs thin film on glass, the inset is the Tauc plot obtained from optical absorbance spectrum. (**e**) Energy level diagram of Au-ZnO-FeS_2_-CuI-ITO p-i-n photodiode before contact, which comes from UPS analysis and band gap of each material, and after contact, resulting in same E_f_ level of three layers.

Moreover, the intrinsic active layer normally has a bandgap which is smaller than both homogeneous regions. The small bandgap of the intrinsic layer determines the long wavelength cutoff of the photoresponse, λ_l_, and the homogeneous region with wide bandgap can serve as window for light to enter, which sets the short wavelength cutoff of the photoresponse, λ_s_. We performed optical absorbance measurement to obtain Tauc plot to determine the band gap of FeS_2_ NCs film. From Fig. 4d, the band gap is about 1 eV, which is consistent with previous report. 3Furthermore, the valence band maximum (VBM), conduction band minimum (CBM) and Fermi energy (E_f_) could be obtained by ultraviolet photoelectron spectroscopy (UPS) measurement. From Fig. 4b, combined with the band gap, the CBM of FeS_2_ NCs film is 3.9 eV, which agrees with the results got by cyclic voltammetry measurement^43^. So the VBM is 4.9 eV which is 1 eV higher than that due to narrower band gap in our case. Similarly, the CBM and VBM of ZnO and CuI can also be obtained from Fig. 4a,c, respectively, which is quite close to previous report^30^. Finally, the VBM, E_f_ and CBM are summarized in Fig. 4e. When heterojunction is formed, the E_f_ of three materials will stay at the same level. It can infer that the photogenerated electrons and holes can be easily transferred to ZnO and CuI respectively due to very close CBM between ZnO and FeS_2_ and VBM between CuI and FeS_2_.

**Figure 5:**
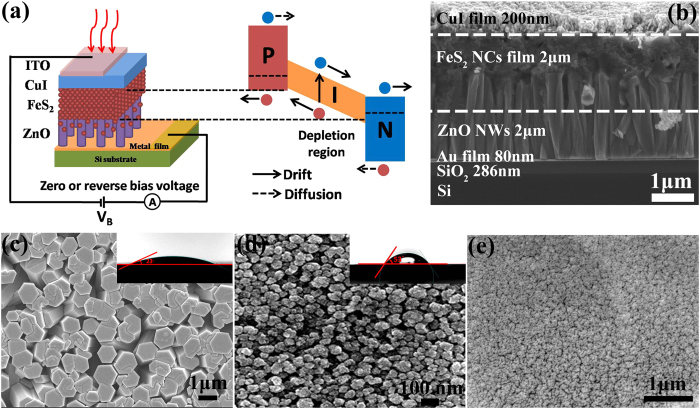
(**a**) Schematic diagram of ZnO-FeS_2_-CuI p-i-n photodiode. The device can work at zero or reverse bias voltage. The drift and diffusion process of electron and hole are shown here. SEM images of typical photodiode device: (**b**) cross section, the thickness of each layer is shown here. Plane view of each layer (**c**) ZnO micro/nanowire arrays, (**d**) FeS_2_ NCs film, (**e**) CuI film. The insets of (**c**) and (**d**) are corresponding water contact angles.

The schematic diagram of ZnO-FeS_2_-CuI p-i-n device is shown in Fig. 5a. When the reverse bias of V_B_ is applied, the width of depletion layer can increase, making this voltage drop falls into the depletion layer. Under the illumination, the photogenerated electrons and holes are swept away by drift in the depletion region and are collected by diffusion from the undepleted region. Therefore, the photogenerated electrons and holes can be extracted from ZnO micro/nanowire arrays by electron selective and CuI film by hole selective contact, respectively. Fig. 5b shows the cross-sectional SEM images of typical ZnO-FeS_2_-CuI p-i-n photodiode. The ZnO micro/nanowire arrays with the length of 2 μm are coated with FeS_2_ NCs film totally, and about 1 μm contact layer provides high surface area of ZnO-FeS_2_ heterojunction. We can see the continuous infiltrating FeS_2_ NCs clearly. At the same time, the compact bottom of ZnO micro/nanowire arrays can hinder the FeS_2_ NCs to contact with the bottom electrode. This 3D heterojunction configuration is quite important for photovoltaic and photodetector device based on 1D ZnO NWs arrays to employ their large surface area to improve heterojunction interfacial area, and direct transport electron to the cathode, reducing the probability of electron back-transfer and recombination^46^. After that, FeS_2_ NCs film is coated by a 200 nm thick CuI layer to create FeS_2_-CuI heterojunction. In particular, we find that the surface wettability plays a key role in building multilayers heterojunction based on solution processed film method. The homogenous and pinhole-free FeS_2_ NCs film and CuI film can be seen from Fig. 5d and 4e, respectively, depending on good surface wettability of each previous layer, seen from insets in Fig. 5c,d. Besides, in Fig. S6 and S7, we have a detailed discussion about the great impact of surface wettability of ZnO micro/nanowire arrays on the morphology of coated CuI film, which directly determines the performance of final photodiode device.

### Photoresponse property

**Figure 6:**
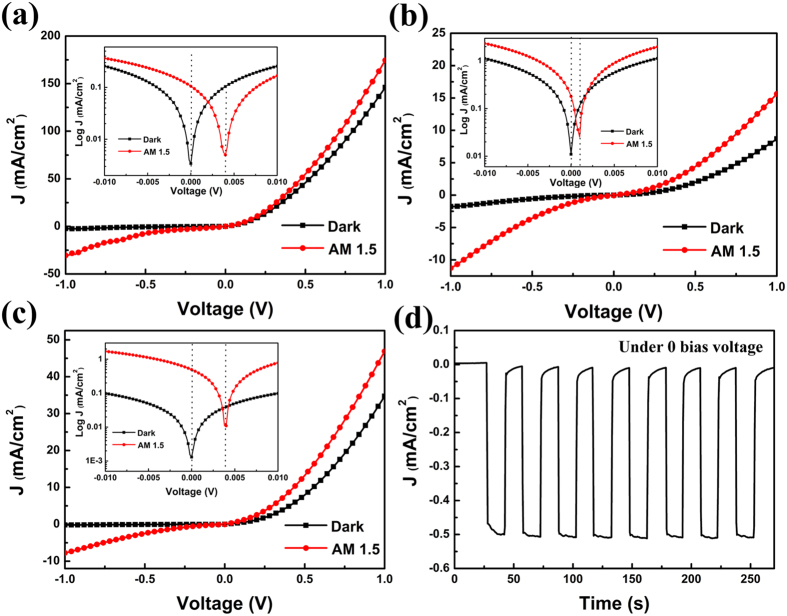
J-V curves obtained under the condition of dark and AM 1.5 for three kinds of devices with Au electrode: (**a**) ZnO-CuI p-n junction, (**b**) ZnO-FeS_2_ junction, (**c**) ZnO-FeS_2_-CuI p-i-n junction. The insets of them are corresponding magnified J-V curve in semi-logarithmic scale near zero bias voltage. (**d**) Time-dependent photoresponse of ZnO-FeS_2_-CuI p-i-n photodiode under dark and AM 1.5 condition.

After this p-i-n heterojunction is formed, a photodiode with good rectifying characteristic is expected. As a comparison, ZnO-CuI and ZnO-FeS_2_ heterojunction device were also prepared to demonstrate the extension of the depletion region as the superiority of p-i-n heterojunction device. Fig. 6a, b, and c show current density-voltage (J-V) characteristics of ZnO-CuI p-n, ZnO-FeS_2_ and ZnO-FeS_2_-CuI p-i-n photodiodesunder the dark and AM 1.5 condition. We can obtain the lowest rectification ratio of 5 at a bias of ±1 V from ZnO-FeS_2_ photodiode. This low rectification ratio is probably caused by a relatively large leakage current or recombination losses due to surface trap states existed in FeS_2_ NCs film. However, the highest rectification ratio of 197 can be obtained from typical diode rectifying curve of ZnO-FeS_2_-CuI p-i-n photodiode. The high CBM of CuI layer can act as an effective electron-blocking layer to reduce back recombination. On the other hand, recently, the PbS NCs solar cell has obtained stable and record power conversion efficiency in air attributing to halide treatment resulting in surface traps passivation^47^,^48^. To some extent, the iodine from CuI layer used here is probably playing a role in passivating surface defect at the interface between FeS_2_ NCs film and CuI film. Under the illumination, the photoresponse can be seen at the forward, reverse and zero bias conditions. The insets of Fig. 6a,b,c show magnified J-V curve near zero bias voltage. The ZnO-FeS_2_-CuI p-i-n photodiode has a large short-circuit current density J_sc_ of 0.5 mA/cm^2^ and a small open-circuit voltage V_oc_ under the AM 1.5 condition. This property makes our p-i-n photodiodes to be feasible for working in a self-power mode^49^, which has been demonstrated in other photodiodes such as ZnO-Spiro-MeOTAD heterojunction^50^, ZnO-CuSCN heterojunction^51^, and ZnO-PbS heterojunction^52^. The on-off current ratio at zero bias voltage is 31, 16, and 371 for ZnO-CuI, ZnO-FeS_2_ and ZnO-FeS_2_-CuI p-i-n photodiodes, respectively. Besides, Fig. 6d shows the repeatable photoresponse of ZnO-FeS_2_-CuI photodiode, and it is found that response and recovery times are shorter than 1 s. The largest on-off current ratio and fast photoresponse of ZnO-FeS_2_-CuI photodiode indicate the structure superiority of p-i-n configuration. From Figure S8, we can see the ZnO-FeS_2_-CuI photodiode with Ag as bottom electrode shows similar results. Even though, a lot of work is necessary to get the optimized thickness of each layer in p-i-n structure to achieve the best performance. Ko *et al’s.* research^30^ on ZnO-PbSe-CuI p-i-n structure indicated the J_sc_ increased with intrinsic layer at the beginning due to increased light absorption and the efficient charge extraction resulted from the assistance of built-in electric field spanning the entire absorber layer. However, the field-free quasi-neutral region could form when the thickness of intrinsic layer was beyond the optimized value, which can increase the recombination probability of photogenerated carriers through this region^53^,^54^. In our case, the low J_sc_ is attributed to formed quasi-neutral region due to micrometre-thick FeS_2_ NCs film. A large number of trap states existed in the FeS_2_ NCs film is believed to cause the carrier recombination, resulting in a very low V_oc_. The notion that halide treatment may be an effective method to overcome this trap states in FeS_2_ NCs confused people for many years.

**Figure 7:**
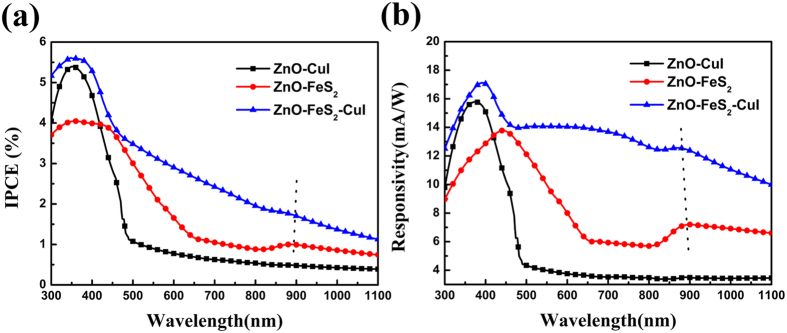
(**a**) IPCE spectrum of three kinds of photodiode under zero bias voltage. (**b**) Wavelength-dependent responsivity spectrum obtained from R = IPCE*λ/1240. When IPCE = 100%, λ = 1240 nm, R = 1 A/W.

The incident photons to current conversion efficiency (IPCE) spectra under zero bias voltage of three devices are shown in Fig. 7a. The ZnO-CuI photodiode shows the maximum IPCE at 350 nm, and IPCE value almost does not change at wavelengths longer than 500 nm, whichmatches the band gap of ZnO and CuI. For ZnO-FeS_2_ and ZnO-FeS_2_-CuI photodiode, the IPCE values increase with decreasing wavelength, which agrees with the absorption spectrum of FeS_2_ NCs film. In order to study the spectral response, we calculated wavelength-dependant responsivity R(λ) in Fig. 7b. The ZnO-FeS_2_-CuI p-i-n photodiode has larger responsivity in the full wavelength region than ZnO-FeS_2_ photodiode, resulting from higher charge collection probability of photogenerated carriers due to wide depletion region. The maximum responsivity is 17 mA/W at 400 nm for ZnO-FeS_2_-CuI p-i-n photodiode, which is comparable with that of ZnO/Ag NWs/ZnO composite film UV photodetector prepared previously^4^. Besides, there is a broad peak shoulder around 900 nm, which is corresponding to optical absorption characteristic of FeS_2_ NCs film. The photoresponsivity of device is extended to near IR wavelength showing the potential as infrared photodetectors applications.

## Conclusion

We have developed a seedless method to grow high density and vertically aligned ZnO micro/nanowire arrays on metal films based on hydrothermal method under the saturated nutrition solution. In particular, the ZnO micro/nanowire arrays have low defect density without atmosphere annealing treatments. A thin film formed from nucleation layer as the buffer layer may release the elastic strain resulted from the lattice mismatch between ZnO and substrate. Besides, the substrate-dependent property of ZnO micro/nanowire arrays indicates that vertically aligned property has a large relationship with wettability and surface nucleation energy of substrate. Moreover, we find that the good surface wettability of ZnO micro/nanowire arrays is the premise of coating homogenous and pinhole-free FeS_2_ NCs film and CuI film to create heterojunction, determining the performance of final photodiode device directly. Based on the ZnO micro/nanowire arrays, we prepared ZnO-CuI p-n, ZnO-FeS_2_ and ZnO-FeS_2_-CuI p-i-n photodiodes. Among them, ZnO-FeS_2_-CuI p-i-n photodiode has the highest rectifying ratio of 197 at ±1 V, resulted from CuI layer that acts as an effective electron-blocking layer to reduce back recombination. Under AM 1.5 condition, the J_sc_ of 0.5 mA/cm^2^ and on-off current ratio of 371 at zero bias voltage are achieved, indicating the feasibility of self-power mode. The maximum responsivity is 17 mA/W at 400 nm, and high responsivity around 900 nm shows the potential for near infrared photodetectors applications. This good performance is attributed to the efficient extraction of photogenerated electrons and holes due to the built-in electric field in increased depletion layer width for p-i-n structure. Finally, the reason for low V_oc_ could be carrier recombination from the trap states existed in the FeS_2_ NCs film due to defects. These defects may be passivated by halide treatment, which has been demonstrated an effective method for Pb chalcogenide.

## Methods

### Fabrication of n-type ZnO electron transport layer

5 nm of Cr and 80 nm of Au/Ag deposited on Si substrate with 286 nm of SiO_2_ layer by thermal evaporation were used to grow ZnO micro/nanowire arrays, and also as bottom electrode of total device. Before growth, the above metal thin films were treated with argon plasma for 10 min to produce good hydrophilic surface. Typically, the growth solution was prepared as follows. 80 mM Zn(NO_3_)_2_ and HMTA were dissolved in deionized water with magnetic stirring for 15 min at room temperature. Then nutrition solution was stood for 5 min to get clear supernatant solution, which was saturated nutrition solution at room temperature. Then the ZnO micro/nanowire arrays with very high density were hydrothermally grown on metal thin films by immersing above substrates floating in saturated nutrition solutions at 80 °C for 7 h in sealed glass bottle. Besides, the growth solution with different concentrations was also investigated to get ZnO micro/nanowire arrays with different density. After growth, the samples were rinsed with deionized water and dried in a nitrogen gas flow.

### Fabrication of ZnO/FeS_2_/CuI p-i-n photodiode

The FeS_2_ NCs were prepared according to previous report^55^. Before depositing FeS_2_ NCs thin film, the FeS_2_ NCs were further purified by centrifugation, and dispersed into hexane to produce NCs solution about 50 mg/ml. Then the FeS_2_ NCs solution was spin-coated on ZnO micro/nanowire arrays at 1000 rpm for 30 s to make the NCs thin film. Next, the thin film was dipped in 0.1 M thioglycolic acid (TGA) in acetonitrile solution for 2 min to conduct ligand exchange, followed by a rinse with acetonitrile to remove the original ligand. This procedure was repeated several times to get desired thickness. After this, the 3 M CuI di-n-propyl sulfide solution was spin-coated on FeS_2_ NCs thin film to produce p-type hole transport layer, which were dried at 120° C for 10 min to make sure the good crystalline of CuI thin film. Finally, the ITO glass was fixed on the CuI thin film acting as top transparent electrode. The active area was confirmed as 0.1 cm^2^.

### Characterization

SEM images were obtained from field-emission JEOL JSM-7001F. TEM images were obtained from JEOL JEM-2100. XRD measurement was carried out with a Rigaku D/max 2400 using Cu Ka radiation. UV-Vis-NIR optical absorption was performed on PerkinElmer Lambda 950 spectrophotometer. The XPS and UPS were measured in a homemade UHV system with the base pressure at 3 × 10^−10^ Torr. A hemispheric electron analyzer (Omicron, EA125) was used to detect the photoelectron excited by a monochromatic Al Kα radiation (hν = 1486.7 eV) or UV light (He I, hν = 21.2 eV). PL spectrum of ZnO micro/nanowire arrays was collected using a spectrometer (Horiba-JY T64000). The excitation source was 325 nm laser with intensity of 0.5 mW, and integration time was 1 s. The surface wettability was measured using a contact angle meter (OCA20, Dataphysics Co., Germany). For photoresponse test, the current-voltage measurements were conducted on Keithley 2400 source meter under illumination by an AM 1.5 G solar simulator (Sciencetech Inc., SS-150). The IPCE was measured by the solar cell quantum efficiency measurement system (Solar Cell Scan 100, Zolix instruments. Co., Ltd.).

## Additional Information

**How to cite this article**: Yang, Z. *et al.* Developing Seedless Growth of ZnO Micro/Nanowire Arrays towards ZnO/FeS_2_/CuI P-I-N Photodiode Application. *Sci. Rep.* **5**, 11377; doi: 10.1038/srep11377 (2015).

## Supplementary Material

Supplementary Information
