# Supplementary material for: Developing Seedless Growth of ZnO Micro/Nanowire Arrays towards ZnO/FeS2/CuI P-I-N Photodiode Application
Source: Sci Rep. 2015 Jun 16;5:11377. doi: 10.1038/srep11377 (PMC4468464; doi:10.1038/srep11377)
Supplement: Supplementary Information [file srep11377-s1.doc]

Supporting Information

**Developing Seedless Growth of ZnO Micro/Nanowire Arrays towards ZnO/FeS_2_/CuI P-I-N Photodiode Application**

*Zhi Yang,^1,2^, Minqiang Wang^1^*^*^*, Sudhanshu Shukla^2,3^, Yue Zhu^2^, Jianping Deng^1^, Hu Ge^3^, Xingzhi Wang^2^, Qihua Xiong^2,4^*^*^

^1^Electronic Materials Research Laboratory (EMRL), Key Laboratory of Education Ministry; International Center for Dielectric Research, Xi’an Jiaotong University, Xi’an 710049, China.

^2^Division of Physics and Applied Physics, School of Physical and Mathematical Sciences, Nanyang Technological University, Singapore 637371, Singapore

^3^Energy Research Institute, Interdisciplinary Graduate School, School of Materials Science and Engineering, Nanyang Technological University, Singapore 639798, Singapore

^4^NOVITAS, Nanoelectronics Center of Excellence, School of Electrical and Electronic Engineering, Nanyang Technological University, Singapore 639798, Singapore

* To whom correspondence should be addressed.

Email: [mqwang@mail.xjtu.edu.cn](mailto:mqwang@mail.xjtu.edu.cn)

Qihua@ntu.edu.sg

1. **Growth of ZnO micro/nanowire arrays on metal thin films by seedless method**

In order to obtain vertical aligned ZnO micro/nanowire arrays with high density, four concentrations were used to study the ralationship between the density and concentration of nutrition solution. At room temperature, the saturated nutrition concentration is around 60 mM. Higher amounts of Zn(NO_3_)_2_ and HMTA will produce flocculate in solution. After a while, the all flocculate drop to bottom, and the clear supernatant is saturated nutrition solution to grow ZnO micro/nanowire arrays. From Figure S1, with increasing concentration of nutrition solution, the number of nuclei increased at the stage of nucleation, resulting in high density of final ZnO micro/nanowire arrays.


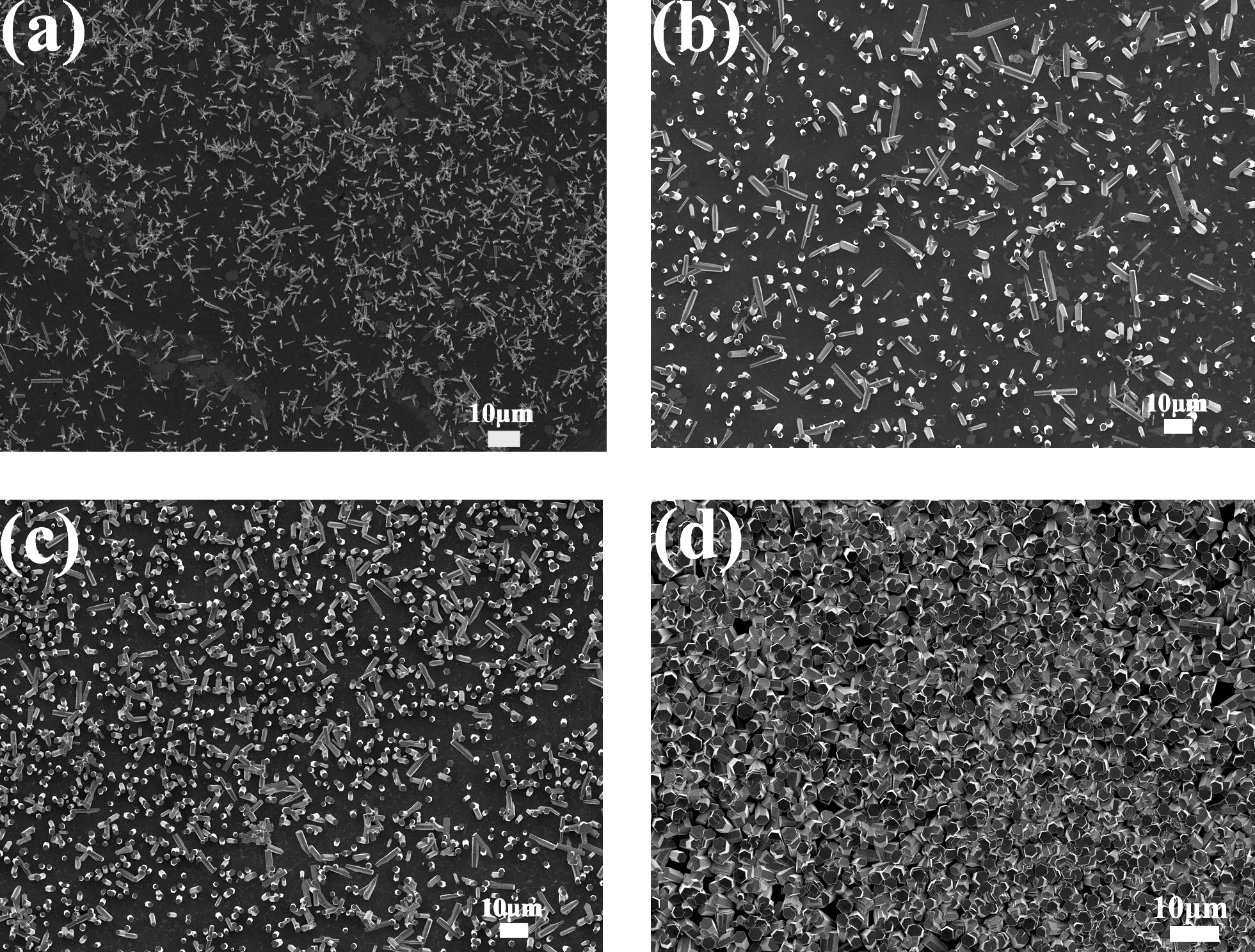


**Figure S1.** SEM images of ZnO micro/nanowire arrays with different density on Au film obtained with diffenrent concentration of nutrition solution: (a) 5 mM, (b) 20 mM, (c)50mM, (d) 100 mM.


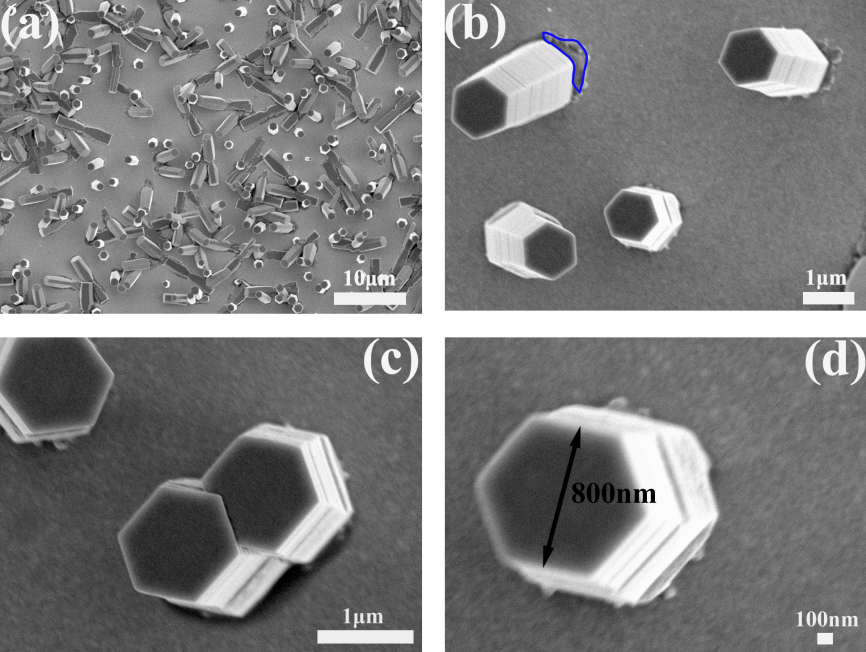


**Figure S2.** SEM images of single ZnO micro/nanowire on Au film with nutrition solution concentration of 20 mM.

From Figure S2, there are many ZnO micro/nanowires laid on the Au film due to isotropic nucleation. Besides, the random orientation appeares for ZnO micro/nanowires grown vertically. From Figure S2b, a thin film appeared between ZnO micro/nanowire and Au film, which is marked by blue circle. There is also cohered micro/nanowire, which support the growth mechanism metioned previously. The diameter of single ZnO micro/nanowire is 800 nm.


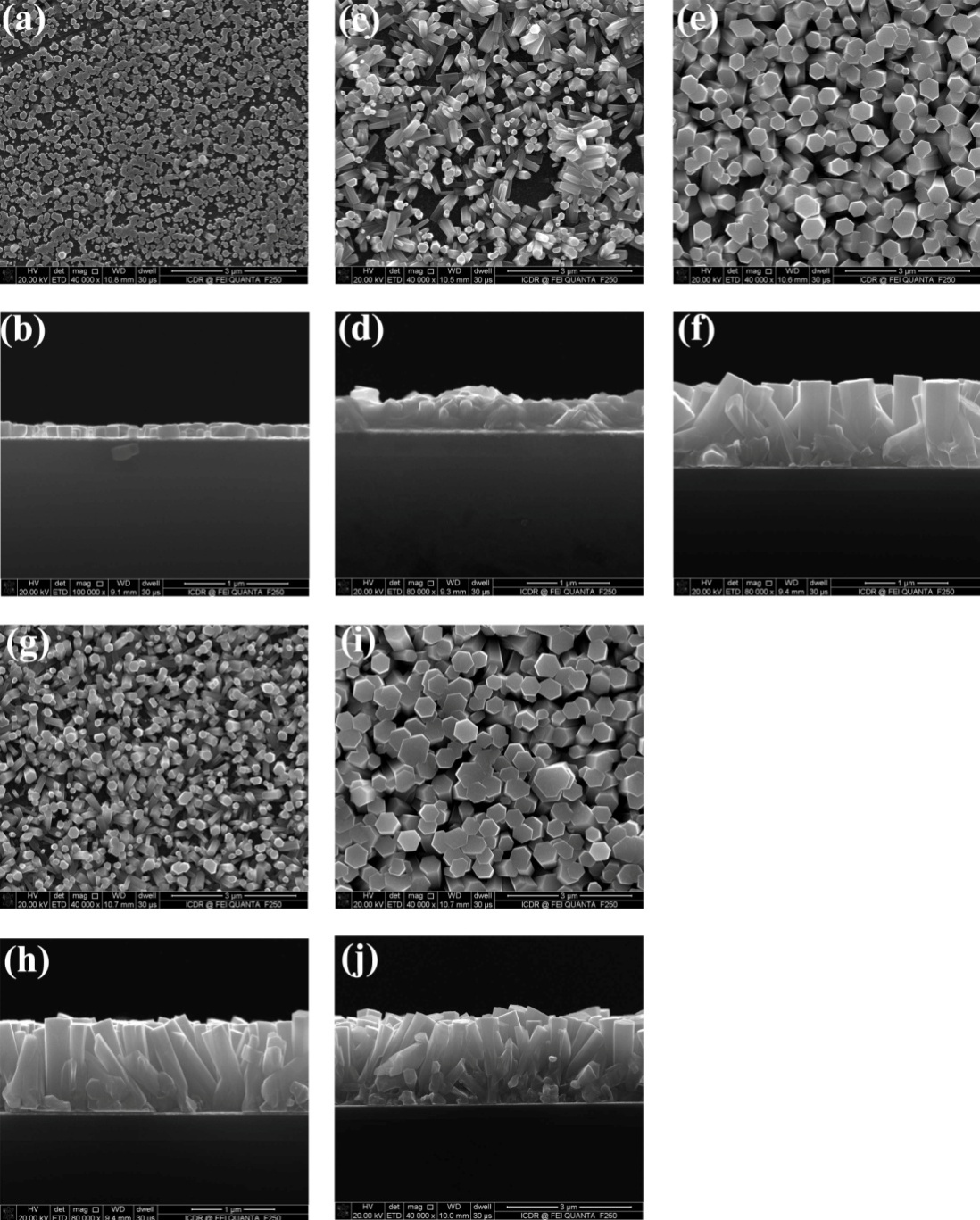
**Figure S3.** SEM images of ZnO micro/nanowires arrays grown on Au film with different growth hours (a,b) 0.5 h, (c,d) 1 h, (e,f) 3 h, (g,h) 5 h, (i,j) 7 h with nutrition solution concentration of 80 mM.

At the 0.5 h, we can seen many vertically aligned ZnO nanowire arrays with 100 nm length, and large number of bare regions on substrate, indicating the nucleation process at the beginning. With the increasing growth time to 1 h, some ZnO nanowires with tilt growth appear and the length keep increasing. At the 3 h, we can see hexagonal ZnO micro/nanowire arrays with high density. At the 5 h, we can see the density of ZnO is increasing, but the length still keeps about 1 um. Finally, longer and more dense ZnO micro/nanowire arrays are obtained.

**
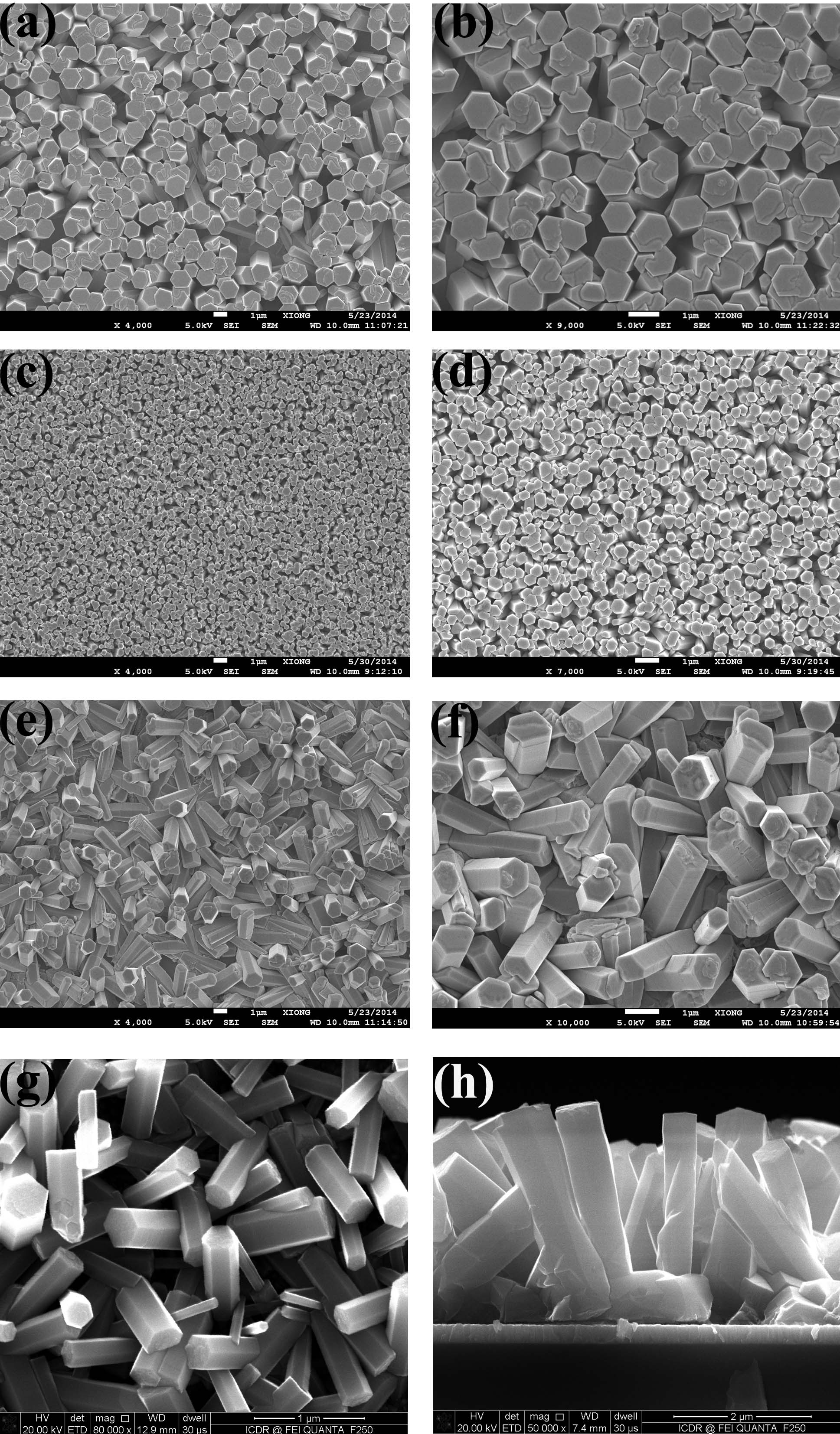
**

**Figure S4.** SEM images of ZnO micro/nanowires arrays grown on (a,b) Au film, (c,d) Ag film, (e,f) graphene, (g) FTO, (h) ITO with nutrition solution concentration of 80 mM. All these subatrates are used directly withour any treatment.


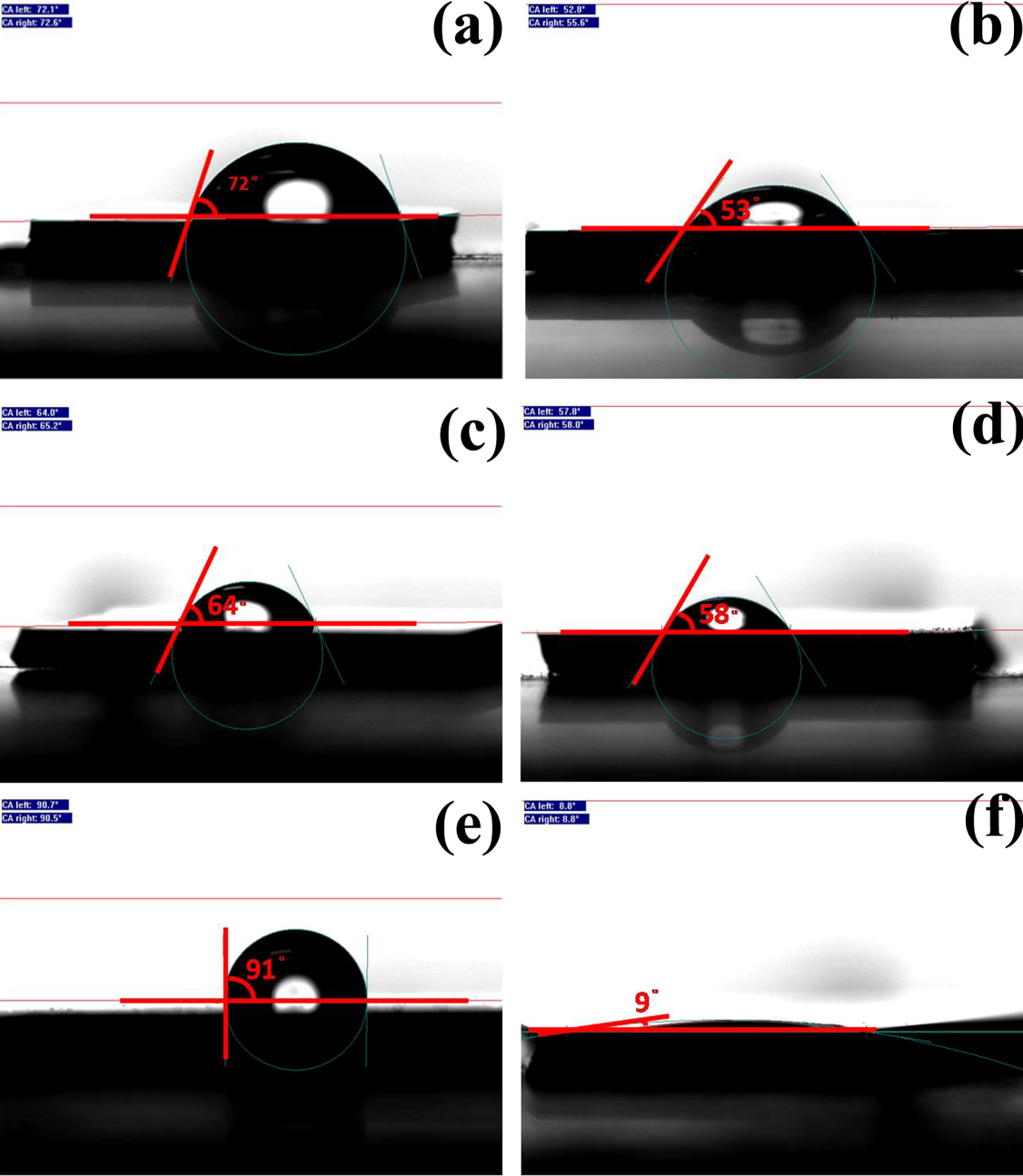


**Figure S5.** Water contact angles of Au film on Si (a) original (b) after Ar plasma treatment; Ag film on on Si (c) original (d) after Ar plasma treatment; graphene on on Si (e) original (f) after Ar plasma treatment.

From Figure S4, the ZnO micro/nanowires arrays grown on Au film and Ag film have better vertically aligned property than graphene, FTO and ITO glasses. From Figure S5, the contact angles mesurements of original substrate indicate that Ag film has the best wettability, and graphene is almost hydrophobic. After Ar plasma treatment, the wettability is improved. However, the graphene on Si substrate has suprisingly excellent wettablity because graphene is removed totally, leaving hydrophilic si substrate. So we think good vertically aligned property is resulted from more flat and hydrophilic surface of metal films. Besides, FTO and ITO glasses have similar or even better wettability compared with metal films. However, they have almost as same aligned property as graphene. We think the final aligned property have a large business with nucleation stage. The metal films may have lower nucleation energy for ZnO, providing more nucleation sites. So higher density ZnO NWs array can be obtained, facilitating vertically aligned property. Even similar surface property for Au and Ag films, the size of ZnO micro/nanowires arrays are different. Compared with Au film, the ZnO micro/nanowires arrays grown on Ag film has smaller diameter, which is attributed to more nucleation at the beginning due to better wettablity or lower nucleation energy of Ag film than Au film. Thus, the ZnO micro/nanowires arrays by seedless method have a large relationship with wettability and surface nucleation energy of substrates. In all, only vertically aligned ZnO micro/nanowires arrays with flat surface are suitable for filling p-type material to create heterojunction.

1. **The morphology and electrical properties of ZnO-CuI p-n photodiode can be improved based on ZnO micro/nanowire arrays with better wettability**

The surface wettability of ZnO micro/nanowire arrays has great impact on the morphology of coated FeS_2_ NCs film and CuI film, which directly determines the performance of final photodiode device. Here, we take ZnO-CuI photodiode for a case to check the impact of wettability on morphology and electrical properties. From Figure S6a, the fresh prepared ZnO micro/nanowire arrays have a water contact angle of 113^。^, which may be caused by the low surface energy (0001) plane at the micro/nanowire surface.^[1,2]^ If the CuI di-n-propyl sulfide solution was spin-coatd on this hydrophobic surface, the discontinuous and self-balling phenomenon appeared in Figure S6b and c , resulting in large leakage for ZnO-CuI-2 photodiode in Figure S7. As contrary, the ZnO micro/nanowire arrays can be transformed to hydrophilic after Ar plasma treatment in Figure S6d. Similar result can be found in literature.^[3]^ The coated CuI film can be compact and continuous in Figure S6e and f, which shows good rectifying characteristic for ZnO-CuI-1 photodiode in Figure S7.


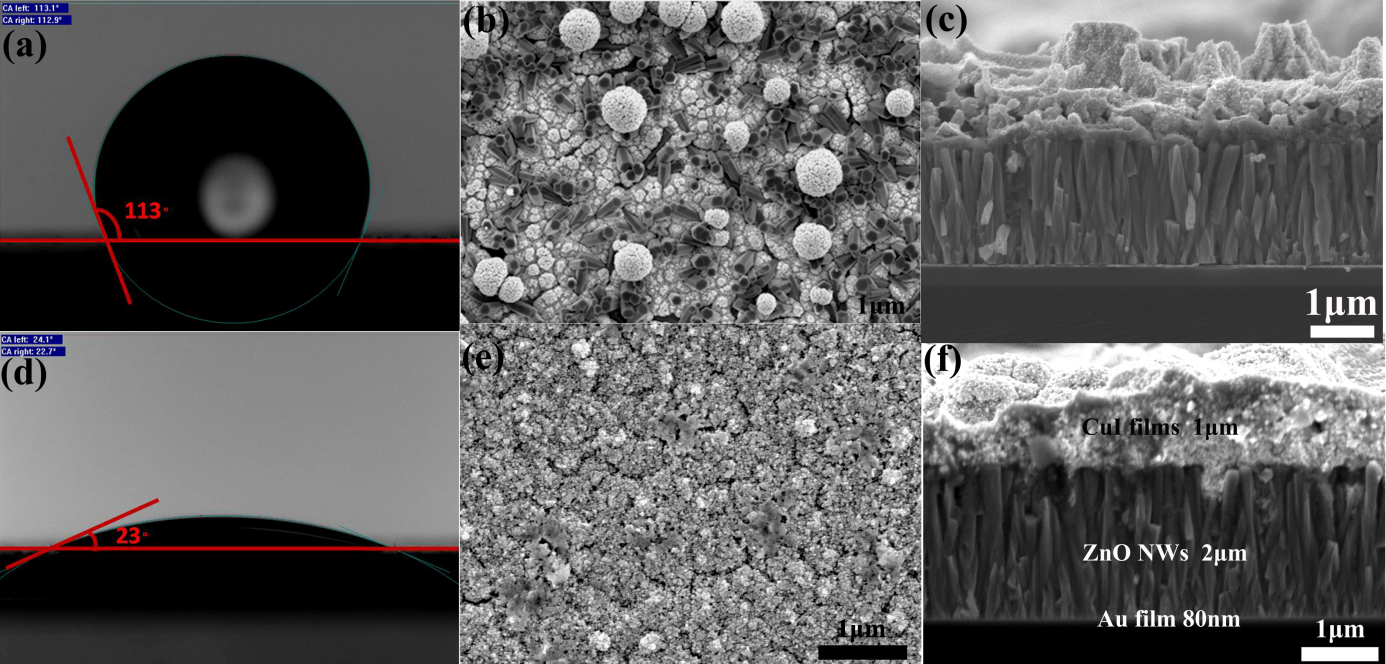


**Figure S6.** Water contact angles of ZnO micro/nanowire arrays (a) fresh prepared and (d) after Ar plasma treatment. (b-c), (e-f) Correspongding SEM images of the ZnO-CuI heterojunction device using ZnO micro/nanowire arrays.


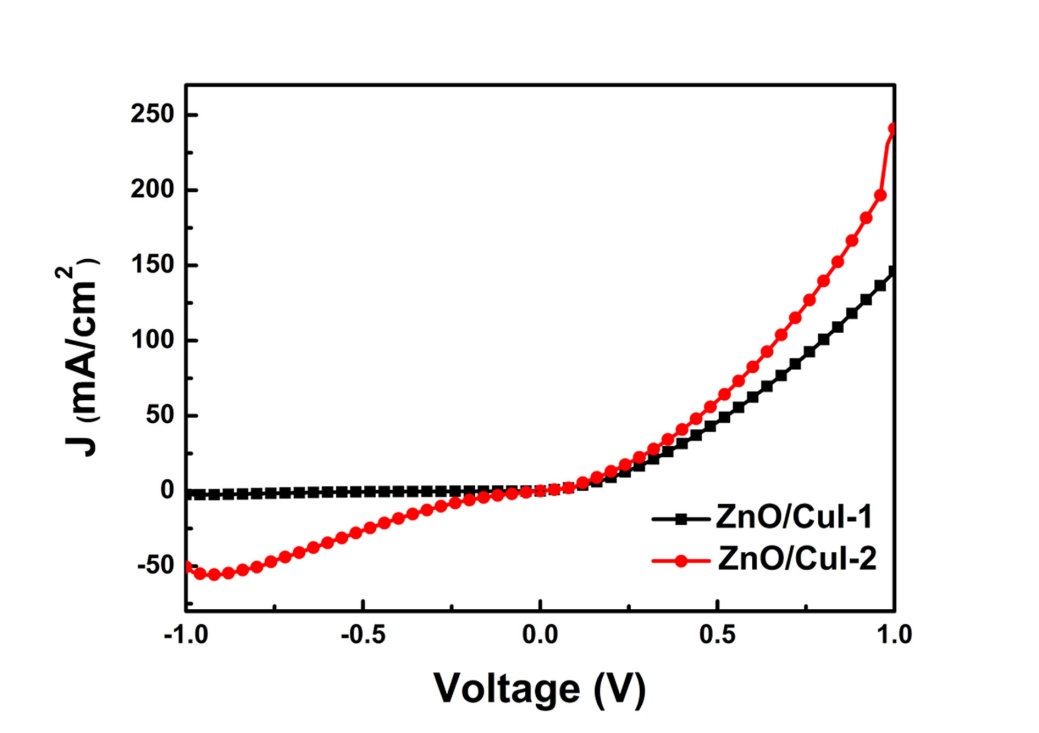


**Figure S7.** The J-V curves of ZnO-CuI-1 photodiode with and ZnO-CuI-2 photodiode without compact coated CuI film under the dark condition.

1. **The photoresposne of ZnO-FeS2-CuI photodiode with Ag electrode**


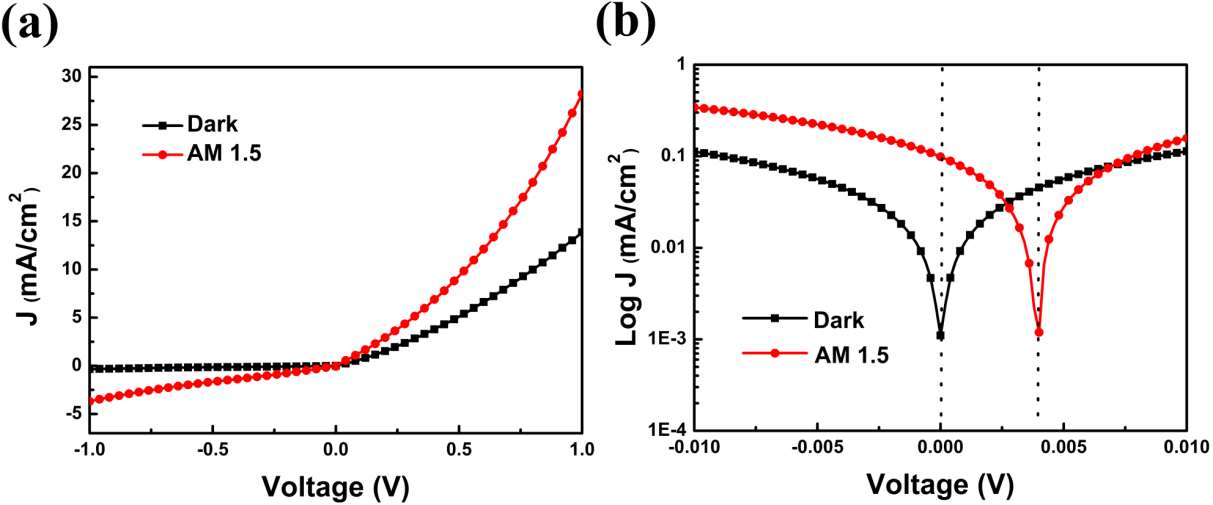


**Figure S8**. The J-V curves of ZnO-FeS_2_-CuI photodiode with Ag electrode under the condition of dark and AM 1.5: (a) linear scale with a wide voltage range, (b) corresponding semi-logarithmic scale graph near zero bias voltage.

**Reference :**

1 H. B. Chen, X. Wu, L. H. Gong, C. Ye, F. Y. Qu, G. Z. Shen, *Nano. Res. Lett*. **2010**, *5*, 570.

2 G. Kenanakis, E. Stratakis, K. Vlachou, D. Vernardou, E. Koudoumas, N. Katsarakis, *Appl. Surf. Sci.***2008**, *254*, 5695.

3 X. Meng, D. Zhao, J. Zhang, D. Shen, Y. Lu, L. Dong, Z. Xiao, Y. Liu, X. Fan, *Chem. Phys. Lett.* **2005***, 413*, 450.
